# Live mucosal vaccination stimulates potent protection *via* varied CD4^+^ and CD8^+^ T cell subsets against wild-type *Brucella melitensis* 16M challenge

**DOI:** 10.3389/fimmu.2022.995327

**Published:** 2022-10-03

**Authors:** Zakia I. Goodwin, Xinghong Yang, Carol Hoffman, David W. Pascual

**Affiliations:** Department of Infectious Diseases and Immunology, College of Veterinary Medicine, University of Florida, Gainesville, FL, United States

**Keywords:** mucosal and systemic immunity to brucellosis *Brucella*, T cells, IFN-γ, IL-17, vaccine, mucosal

## Abstract

Re-emerging zoonotic pathogen *Brucella* spp. continues to impact developing countries and persists in expanding populations of wildlife species in the US, constantly threatening infection of our domestic herds. The development of improved animal and human vaccines remains a priority. In this study, immunity to a novel live attenuated *B. melitensis* strain, termed znBM-mC, was characterized. An oral prime, intranasal (IN) boost strategy conferred exquisite protection against pulmonary challenge, with wild-type (wt) *B. melitensis* providing nearly complete protection in the lungs and spleens from brucellae colonization. Vaccination with znBM-mC showed an IFN-γ^+^ CD8^+^ T-cell bias in the lungs as opposed to Rev 1-vaccinated mice showing IFN-γ^+^ CD4^+^ T-cell inclination. Lung CD4^+^ and CD8^+^ effector memory T cells (TEMs) increased over 200-fold; and lung CD4^+^ and CD8^+^ resident memory T cells (TRMs) increased more than 250- and 150-fold, respectively. These T cells served as the primary producers of IFN-γ in the lungs, which was essential for vaccine clearance and the predominant cytokine generated pre-and post-challenge with wt *B. melitensis* 16M; znBM-mC growth could not be arrested in IFN-γ^−/−^ mice. Increases in lung TNF-α and IL-17 were also induced, with IL-17 being mostly derived from CD4^+^ T cells. Vaccination of CD4^−/−^, CD8^−/−^, and B6 mice with znBM-mC conferred full protection in the lungs and spleens post-pulmonary challenge with virulent *B. melitensis;* vaccination of IL-17^−/−^ mice resulted in the protection of the lungs, but not the spleen. These data demonstrate the efficacy of mucosal vaccine administration for the generation of protective memory T cells against wt *B. melitensis*.

## Introduction

*Brucella* spp. are Gram-negative intracellular bacteria responsible for causing abortion in mammals (1). Over 12 species have been identified, five of which are infectious to humans (2–4). Disease in livestock is primarily caused by three species, namely, *B. abortus, B. melitensis*, and *B. suis*, listed in the Terrestrial Code of the World Organization for Animal Health (OIE) as reportable to the Veterinary Authority (5), and, in endemic countries, brucellosis results in billions of dollars of lost income annually (6, 7). The Centers for Disease Control (CDC) consider these three *Brucella* species as select agents (8). Consequently, infection with either of these poses significant high health care costs for diagnosis, treatment, and management. This, coupled with the low infectious dose and potential for aerosolization, increases the risk of illicit dissemination (8, 9). Although brucellosis is not a new disease and is believed to be in existence since the domestication of livestock, the lack of effective vaccines has renewed efforts to develop more effective animal vaccines and human vaccine (6, 10–12).

Live, attenuated brucellosis vaccines are used for livestock, but none for humans (11–13). The smooth *B. abortus* S19 vaccine is a mutant originally derived from a virulent isolate (14), and is effective in nonpregnant cows. The rifampicin-resistant rough *B. abortus* 51 (RB51) vaccine (15) quickly replaced S19 in many regions, although both are equally effective. RB51 enables distinguishing naturally infected from vaccinated animals (16–18). The streptomycin-resistant *B. melitensis* Rev 1 vaccine is effective against small ruminants (19), and is widely used to protect goats and sheep (20). Despite their use, brucellosis remains problematic worldwide, in part due to their incomplete efficacy, warrants the development of novel replacement vaccines (11–13).

Complementing vaccine development, an optimized vaccination regimen to improve efficacy needs consideration. In fact, while *Brucella* primarily infects the host following a mucosal exposure (21–23), research has largely emphasized parenteral infections due to the ability of *Brucella* to cause systemic infections (24). Most brucellosis vaccine studies fail to consider mucosal immunization as an integral component for optimal vaccine efficacy or host immunity (12). As a result, few studies have investigated vaccination by the nasal or oral routes (25–29). The absence of brucellae or pathology in the intestinal tract (4) suggests that other mucosal sites, such as the oropharyngeal tissues, are responsible for infections (22). Thus, vaccination *via* the oropharyngeal route would be anticipated to mimic natural infection, stimulating immune responses capable of neutralizing brucellae (29, 30).

To date, live attenuated vaccines and vaccine candidates offer the most effective protection against infection (2, 11, 12, 31). Such vaccinations require the stimulation of cell-mediated immunity. In fact, the stimulation of IFN-γ is essential for host protection against *Brucella* infection (21, 32–34). Conventional livestock vaccines, S19 and RB51 for *B. abortus* and Rev 1 for *B. melitensis*, typically induce protective CD4^+^ Th1 cell responses (34–36), but discount the advantages of mucosal vaccination in stimulating alternative immune cell subsets. In this vein, the thrust toward mucosal vaccination has gained traction with promising results (23, 25, 29, 37). Like natural infection, which is controlled early on by CD8^+^ T cells and γ/δ^+^ T cells, consideration of the importance of CD8^+^ T cell-dependent immunity subsets after mucosal vaccination is being recognized as an alternative arm of protection (23, 37, 38).

In this study, a novel *B. melitensis* mutant was developed similar to our *B. abortus* mutant (39, 40). The *ΔznuA ΔnorD B. melitensis* carrying the mCherry as a DIVA (znBM-mC) shows superior protection against challenge with wild-type (wt) *B. melitensis* 16M conferred by induced CD4^+^ and CD8^+^ resident memory T cells (TRMs). Using an oral prime, nasal boost vaccination regimen with znBM-mC protects against pulmonary challenge.

## Materials and methods

### *Brucella* strains

A double mutant on a *B. melitensis* 16M background was constructed similar to that done with the *ΔznuA ΔnorD B. abortus-lacZ* (znBAZ) (40). The *znuA* gene encodes a high-affinity periplasmic binding protein-dependent and ATP-binding cassette (ABC) transport system for Zn^2+^ (39, 41). The *norD* gene, a member of the *norEFCBQD* operon, encodes a nitric oxide reductase (42–44). Because the mutant still bears its LPS, rendering it indistinguishable from naturally infected animals, a means of differentiating infected from vaccinated animals (DIVA) was accomplished *via* the insertion of mCherry (mC) into chromosome I within an uncoded region between BMEI1800 and BMEI1801 under the *Brucella* constitutive promoter, PsojA. This mutant was determined to be sensitive by the absence of growth in the presence of various antibiotics, including ampicillin, doxycycline, gentamicin, kanamycin, rifampicin, chloramphenicol, and streptomycin. The completed double mutant strain is referred to as znBM-mC (*ΔznuA ΔnorD B. melitensis*-mCherry).

Live *Brucella* strains were prepared from working stock and inoculated onto Potato Infusion Agar (PIA) plates. Plates were incubated at 37°C and 5% CO_2_ for 3–5 days before harvesting. Bacteria removed from the plate were washed twice for 10 min in sterile Phosphate Buffered Saline (sPBS) and diluted to the required concentration in sPBS.

### Mice

All animal experiments using live attenuated *B. abortus* strains RB51 and S19 were performed under biosafety level 2 (BSL-2) containment, and those with znBM-mC mutant, Rev 1, and wt *B. melitensis* 16M strains were performed under biosafety level 3 (BSL-3) containment. Female 6–8-week-old mice were used for all experiments. The BALB/c and C57BL/6 mice were purchased from Charles River Laboratory (Frederick, MD, USA). CD4^−/−^, CD8^−/−^, and IL-17^−/−^ mice were purchased from Jackson Laboratory (Bar Harbor, ME). IFN-γ^−/−^ (H-2^b^) mice were bred in-house. Animals were maintained in individually ventilated cages under HEPA-filtered-barrier conditions of 12 h of light and 12 h of darkness and were provided with food and water *ad libitum*. All animal care and procedures were in accordance with institutional policies for animal health and well-being approved by the University of Florida Institutional Animal Care and Use Committee.

### Vaccination and challenge

Various routes of vaccination were tested: oral prime, intranasal (IN) boost, IN only, and lN prime and boost. All priming was done on day 0 and all boosts were performed on day 28. For oral vaccinations, mice were first gavaged with 200 µl of 10% saturated sodium bicarbonate solution using a 20-gauge oral gavage needle attached to a 1 ml luer-lock syringe. After 10 min, mice were then dosed with the bacteria strain diluted to the required concentration in 200 μl volume using the same technique. For IN vaccination, isoflurane-anesthetized mice were given brucellae prepared to the required concentration in a final volume of 30 μl. Each mouse received the vaccine drop into each nostril using a micropipette. Anesthetizing mice and using a high volume (30 μl) of vaccine ensures delivery to the lungs as opposed to the upper respiratory tract. For pulmonary challenge with wt. *B. melitensis* 16M, brucellae were prepared as 5 × 10^4^ CFUs in 30 μl sPBS and administered by the nasal route as done for nasal vaccinations. Serial dilutions of inoculum on PIA plates were assessed to verify vaccination and challenge doses.

For oral prime, IN boost protection studies, groups of mice (n = 5–7/group) were orally primed on day 0 with 10^9^ CFUs znBM-mC, 10^9^ CFUs S19, 10^9^ CFUs Rev 1, or sPBS. On day 28, mice were nasally boosted with 5 × 10^8^ CFUs znBM-mC, 10^9^ CFUs S19, 5 × 10^8^ CFUs Rev 1, or sPBS. On day 56, all mice were subjected to a pulmonary challenge using 5 × 10^4^ CFUs wt *B. melitensis* 16M administered in 30 μl by the nasal route. All vaccination doses were confirmed as described above. On days 70 or 84, individual lungs and spleens were harvested, homogenized in sterile water with Tissue Lyser (QIAGEN, Germantown, MD), and 10-fold serial dilutions were plated on Farrell’s media (Oxoid Ltd., Basingstoke, UK) to measure the extent of wt brucellae colonization. Splenic inflammation was determined by measuring their weights. The experiments were performed two or three times.

For IFN-γ^−/−^ mice, a single oral dose of Rev 1, znBM-mC, or sPBS was administered on day 0, and the study was terminated on day 21. The spleens, lungs, peripheral lymph nodes (PLNs), head and neck LNs (HNLNs), and lower respiratory LNs (LRLNs) were harvested to measure the extent of brucellae colonization.

### T-cell analyses

Mice were orally primed, IN boosted as previously described, and on day 56 (28 days post-boost), individual spleens and lungs were collected in 2 ml tubes containing 1 ml of incomplete media (ICM): RPMI 1640 (Caisson Labs, Inc, Smithfield, UT); 10 mM HEPES buffer (Caisson Labs); and 10 mM penicillin/streptomycin (Caisson Labs); and a single, sterile bead (McMaster-Carr, Elmhurst, IL). Tissues were homogenized (Tissue Lyser; QIAGEN), then filtered through 70 µm disposable cell strainers (Fisherbrand) and washed with ICM at 1,500 RPM for 5 min at 4°C. Splenic red blood cells were lysed with 5 ml of ammonium-chloride-potassium (ACK) buffer: 0.15 M NH_4_Cl, 10 mM KHCO_3_, 0.1 mM Na_2_EDTA) for 5 min. Lung homogenates in 2 ml ICM were digested with 20 µg of Liberase TL research grade (Roche Life Science, Indianapolis, IN) and 50 units of RNase-free DNase I (Promega Corp., Madison WI), incubated at 37°C under 5% of CO_2_, with gentle shaking for 45 min. Digestion was stopped by the addition of 5 μl of 0.5M EDTA for 5 min. Lung homogenates were then filtered through 70 μm disposable cell strainers, washed with ICM, and the remaining red blood cells were lysed with ACK buffer for 3 min at room temperature. After the final wash with ICM, lung and splenic lymphocytes were resuspended in complete media (CM): ICM plus 10% fetal bovine serum (Atlanta Biologicals, Norcross, GA), 10 mM nonessential amino acids (Caisson Labs), and 10 mM sodium pyruvate (Caisson Labs).

Splenic and lung lymphocytes were enumerated using a Cellometer Auto Cell Counter (Nexelcom, Bioscience). Lymphocytes were then cultured overnight at 37°C and 5% CO_2_ at a concentration of 2 × 10^6^ cells/ml in 48-well Corning Costar plates (Sigma-Aldrich, Inc. St. Louis, MO) with stimulation as follows; 10^9^ CFUs/ml heat-killed RB51 (*Brucella* antigen [Ag]) followed by 4–6 h of 5 ng/ml phorbol myristate acetate (PMA) (Sigma-Aldrich) and 500 ng/ml ionomycin (Sigma-Aldrich). Brefeldin A (10 μg/ml; eBioscience, San Diego, CA) was also added during the 4–6 hour stimulation to block cytokine secretion. Stimulated cells were then stained with fluorescently labeled monoclonal antibodies (mAbs) against T-cell surface antigens and cytokines.

### *In vivo* antibody labeling

Resident T cells were distinguished from circulating (vascular) T cells in the lungs by injection *via* the retro-orbital route with 100 µl of Percp-coupled anti-CD45 mAb (2.5 µg/ml; clone YW62.3, eBioscience) and/or PE-Cy7-coupled anti-CD8 mAb (10 µg/ml; clone 53–5.8, eBioscience). Mice were euthanized 10 min after injection. After lymphocyte isolation, lymphocytes were stained *ex vivo* with a different, noncompeting mAb clone of anti-CD45 (clone 30-F1) or anti-CD8 (clone 53-6.7, Thermo-Fisher Scientific) mAb in addition to fluorochrome-conjugated mAbs for other cell surface markers. Circulating memory T cells were distinguished as being intravenous (IV) CD45^+^, whereas noncirculating (resident) memory T cells were IV CD45^−^. Stained cells were analyzed by flow cytometry analysis.

### *In vitro* antibody staining and flow cytometry

To identify T-cell subsets induced by *Brucella* strains used for vaccination and challenge, restimulated lymphocytes were washed with FACS buffer (dPBS with 2% FBS) and then labeled with mAbs specific for TCRβ, CD45, CD4, CD8a, CD44, CD62L, CD69, CD103, CD19, and γδ-TCR for 20 min on ice. After washing in FACS buffer, cells were fixed with IC Fixation Buffer (eBioscience, San Diego USA), permeabilized with 1× Permeabilization Buffer (eBioscience, San Diego USA), and stained for intracellular markers with mAbs specific for IFN-γ, TNF-α, and IL-17. Fluorescence was acquired using a BD Fortessa flow cytometer with the BD FACSDiva software. All the fluorescently conjugated mAbs were purchased from Biolegend (San Diego, USA) or eBioscience (San Diego, USA). Analysis was performed using FlowJo software (TreeStar, Ashland, OR, USA).

### Statistical analysis

One-way analysis of variance (ANOVA) or two-way ANOVA with multiple comparison tests were used to compare groups depending on the data sets. GraphPad Prism 9 version 9.3.1 (471) for Windows, GraphPad Software, San Diego, California, USA (www.graphpadprism.com) was used to determine statistical significance and generate graphs.

## Results

### Mucosal vaccination with znBM-mC confers nearly complete protection in the lungs and spleens after pulmonary challenge with *B. melitensis* 16M

**Figure 1:**
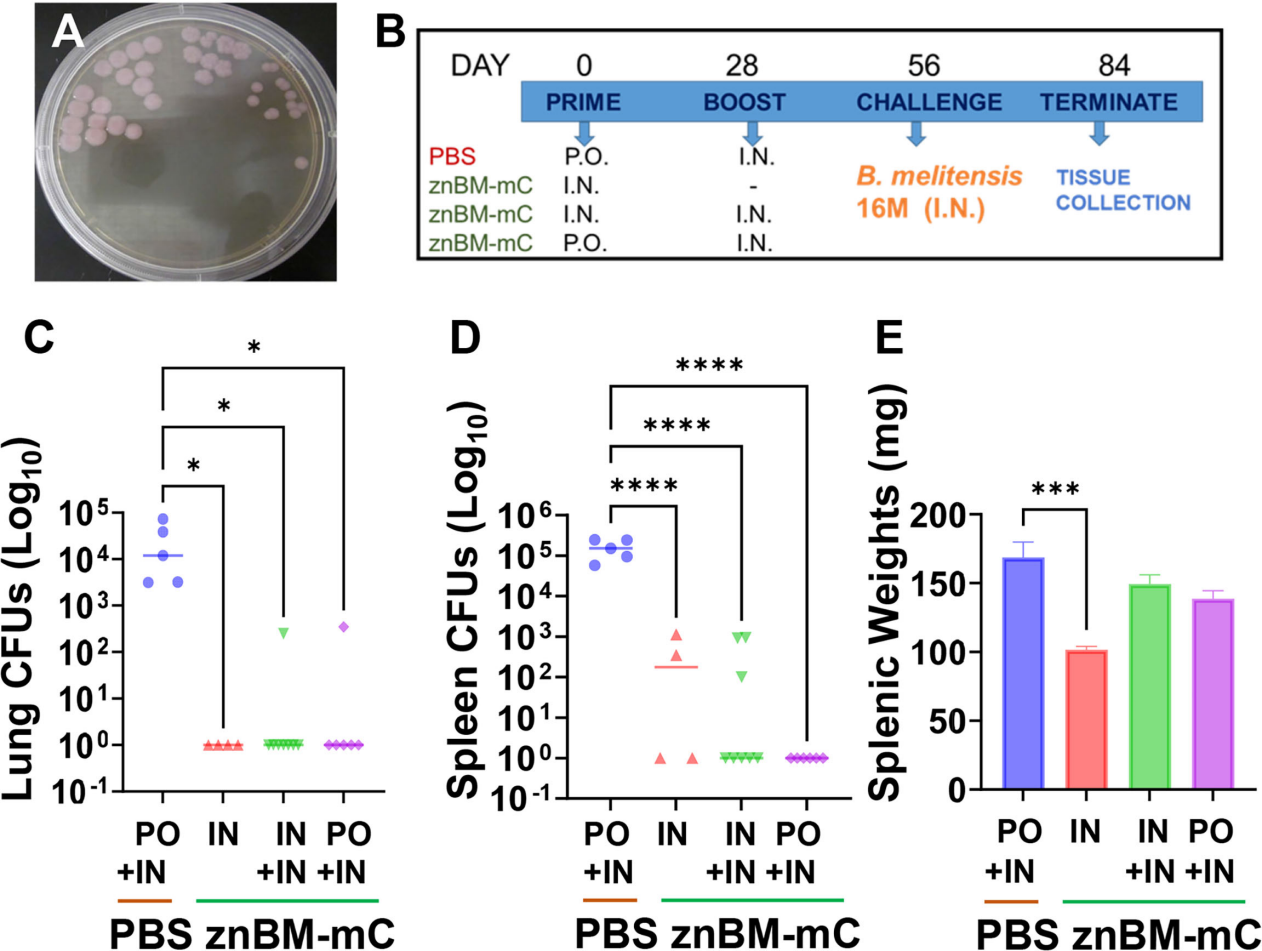
Oral prime, IN boost with znBM-mC confers potent protection against challenge with virulent *B. melitensis* 16M. **(A)** The znBM mutant was generated to express mCherry (noted as pink colonies on PIA) to readily distinguish znBM-mC from other *Brucella* strains. **(B)** Four groups of BALB/c mice were orally or IN vaccinated on day 0 with 10^9^ CFUs znBM-mC, and controls were orally dosed with sterile PBS (sPBS) vehicle. On day 28, one oral and one IN groups were IN boosted with 10^9^ CFUs znBM-mC; one nasal group was left unboosted and the PBS group was IN boosted with sPBS. On day 56, all mice were subjected to pulmonary challenge with 5 × 10^4^ CFUs wt *B. melitensis* 16M. On day 84, individual lungs and spleens were collected for CFU enumeration. **(C)** All znBM-mC vaccinated mice showed a significant decrease in lung colonization compared to naïve controls. Mice receiving a single IN dose show sterile protection. **(D)** All znBM-mC-vaccinated mice showed a significant reduction in splenic colonization compared to naïve controls, particularly, the oral primed, IN boosted mice, which showed sterile protection in the spleens. **(E)** All vaccinated mice showed modest inflammation of the spleens, but those given a single IN dose of znBM-mC displayed significant reduction in splenic weight. Data depict results from five to seven mice per group; *p <0.05, ***p <0.0005, and ****p <0.0001.

The znBM-mC mutant was developed by sequential deletions of *znuA* followed by *norD* genes from the wt *B. melitensis* strain, similar to that done with *B. abortus* 2308 (40). The loss of *znuA* significantly attenuates the capacity of *Brucella* to replicate in the absence of Zn^2+^ acquisition and is cleared within 8 weeks of initial infection (39). To help expedite the clearance of the mutant, a second deletion was introduced into the *norD* gene, which encodes for a nitric oxide reductase (42–44). As done for znBAZ, these mutations are stable and significantly attenuate *Brucella* (40). Since znBM retains its LPS, the DIVA, mCherry (mC), was introduced into a site believed to be inconsequential to its virulence using an endogenous promoter, PsojA. The mC expression readily distinguishes the mutant strain as being notably pink (**Figure 1A**). Hence, the znBM-mC was generated and tested for its ability to elicit protective immunity.

To determine the optimal route for vaccination, various routes and combinations were tested. Preliminary experiments revealed a transient lethargy and rough coat when equivalent doses of prime and boost were administered; hence, a reduced dose for boosting alleviated this reaction (**Figure 1B**). Three groups of BALB/c mice (6–10 mice/group) were vaccinated by different routes: two groups were given a single IN prime on day 0 with znBM-mC and the third group, oral znBM-mC. The control group was given an oral dose of sPBS only. On day 28, one group of IN mice received no further treatment, while the remaining two groups were IN boosted with znBM-mC. The vehicle control group received a nasal sPBS. Four weeks after the boost, on day 56, all groups of mice were subjected to a pulmonary challenge with virulent *B*. *melitensis* 16M. Four weeks later, lungs and spleens were harvested for brucellae enumeration (**Figures 1C, D**). All vaccinated groups showed a significant reduction in colonization in both the lungs and spleens (**Figures 1C, D**); however, the oral prime-nasal boost-treated mice proved the best, with 83% and 100% of mice showing complete protection against wt *B. melitensis* 16M in the lungs and spleens, respectively. Although the single, IN dosed mice showed exquisite protection in the lungs, some reduced colonization of the spleen did remain, and further IN boost with a second dose did not reduce colonization (**Figure 1D**). As an indicator of inflammation, splenic weights were also measured. While all vaccinated groups showed a significant reduction in splenic weights compared with the PBS-dosed controls, the treatment group that received a single IN dosed group showed a marked decrease in spleen weight (p <0.005) (**Figure 1E**).

### Both mucosal znBM-mC and Rev 1 vaccination induced similar magnitude of protection but znBM-mC induced less splenic inflammation

**Figure 2:**
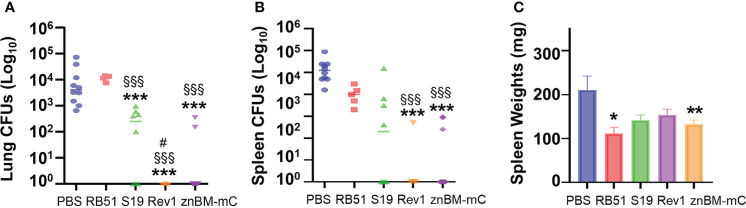
Mucosal vaccination with znBM-mC or Rev 1 elicits protective immunity superior to RB51 or S19 against a wt *B. melitensis* 16M challenge. All groups of BALB/c mice were vaccinated *via* the oral route with 10^9^ CFUs each with *B. abortus* RB51, *B. abortus* S19, *B. melitensis* Rev 1, or znBM-mC on day 0 as described in **Figure 1B**. The vehicle control group was orally dosed with sPBS only. All mice were IN boosted with their respective vaccines on day using 5 × 10^8^ CFUs, and then subjected to pulmonary challenge with 5 × 10^4^ CFUs of wt *B. melitensis* 16M on day 56. On day 84, lungs and spleens were collected, weighed, and homogenized in sPBS with serial dilutions plated on Farrell’s media for CFU enumeration. **(A)** Both Rev 1- and znBM-mC-vaccinated mice showed significant reduction in lung colonization compared to PBS-dosed and RB51-vaccinated mice; Rev 1-vaccinated mice showed significant reduction to S19-vaccinated mice. **(B)** Both Rev 1- and znBM-mC-vaccinated mice showed significant reduction in splenic colonization compared to PBS-dosed and RB51-vaccinated mice. **(C)** Only znBM-mC- and RB51-vaccinated mice showed a significant reduction in splenic weights compared to PBS controls. Data represent two independent experiments with five to seven mice per group; *p <0.05, **p <0.005, and ***p <0.0005 versus PBS-dosed mice; ^§§§^p <0.001 vs. RB51-vaccinated mice; and ^#^p <0.05 vs. S19-vaccinated mice.

To examine the effectiveness of znBM-mC for protection relative to other livestock *Brucella* vaccines, the oral prime-nasal boost regimen was applied to *B. abortus* RB51 and S19, and *B. melitensis* Rev 1 vaccines. Since *B. abortus* and *B. melitensis* share more than 94% DNA homology (3), vaccination with one species can cross-protect against the other species (45). Groups of BALB/c mice were orally primed on day 0 with 10^9^ CFUs RB51, S19, Rev 1, or znBM-mC. A control group of mice was orally dosed with sPBS. On day 28, mice were nasally boosted with 10^9^ CFUs RB51, 10^9^ CFUs S19, 5 × 10^8^ CFUs Rev 1, and 5 × 10^8^ CFUs znBM-mC. Mice were challenged 4 weeks later with wt *B. melitensis* 16M as described previously, and 4 weeks post-challenge, lungs and spleens were harvested for CFU enumeration. Lung colonization post-challenge revealed a significant reduction in colonization by 3-log in znBM-mC-vaccinated mice compared to PBS-dosed controls (p <0.005) (**Figure 2A** **)**. Compared to RB51 and S19-vaccinated mice, lung colonization in znBM-mC dosed mice was also significantly reduced. The znBM-mC-vaccinated group showed improvement in reducing brucellae colonization of the lungs by 41-fold compared with RB51-vaccinated mice. Rev 1- and znBM-mC-vaccinated mice showed equivalent protection in the lungs. Among all vaccinated groups, znBM-mC- and Rev 1-vaccinated mice showed the best reduction in splenic colonization by wt *B. melitensis* 16M with a 4-log reduction (p <0.005) compared with RB51- and S19-vaccinated mice, which showed 1-log and 2-log reduction, respectively, compared to PBS-dosed controls (**Figure 2B**). Wt brucellae colonization of the spleens in RB51- and S19-vaccinated mice was not significant relative to PBS-dosed mice. Mice vaccinated with RB51 and znBM-mC showed a significant reduction in splenic weight compared with unvaccinated PBS controls (**Figure 2C**). Thus, znBM-mC is equivalent to Rev 1 in its efficacy and is less inflammatory.

### Mucosal vaccination with znBM-mC induces both noncirculating and circulating CD4^+^ and CD8^+^ T-cell responses in the lungs

**Figure 3:**
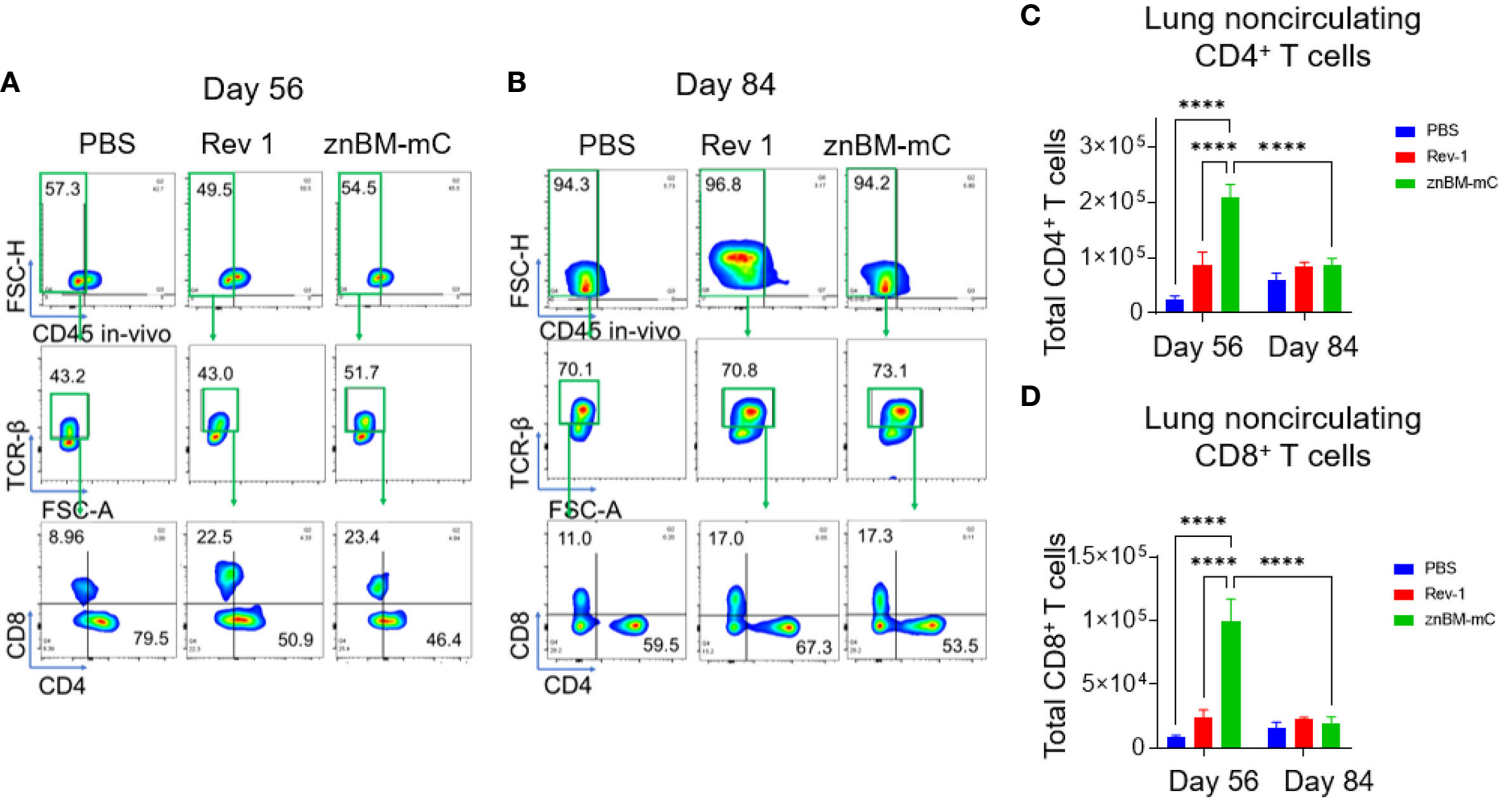
Oral prime, IN boost induces expansion of noncirculating lung CD4^+^ and CD8^+^ T cells, and these are reduced following a *B. melitensis* 16M challenge. Groups of mice were vaccinated with sPBS, Rev 1, or znBM-mC and then subjected to pulmonary challenge with 5 × 10^4^ CFUs wt *B. melitensis* 16M as described in **Figure 2**. On days 56 and 84, lung lymphocytes were isolated and stained for flow cytometry analysis. All mice were given a retroorbital injection of fluorochrome-labeled anti-CD45 mAb 10 min prior to euthanasia. Representative FACS plots of lung T-cell subsets show noncirculating T cells identified as IV CD45^−^ cells on days **(A)** 56 and **(B)** 84, gated as TCRβ^+^ cells, and then gated as CD4^+^ and CD8^+^ T-cell subsets. After vaccination, both groups showed a significant increase in **(C)** CD4^+^ and **(D)** CD8^+^ T cells in the lungs compared to PBS-dosed mice. One-way ANOVA was used for statistical analysis; ****p <0.0001 vs. sPBS-dosed mice.

**Figure 4:**
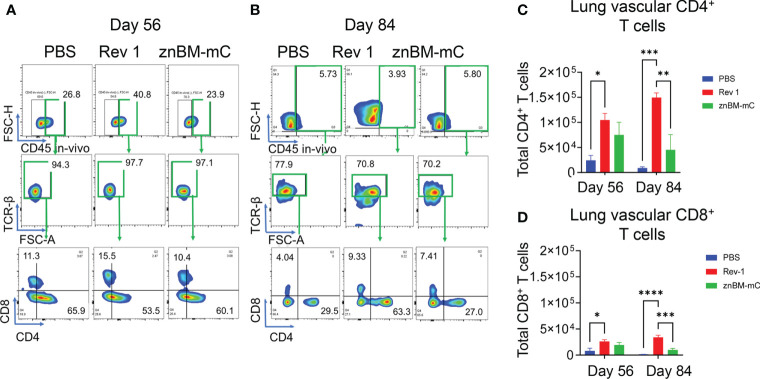
Mucosal vaccination with znBM-mC increases vascular lung CD4^+^ T cells. Groups of mice (the same as described in **Figure 3**) were vaccinated with sPBS, Rev 1, or znBM-mC and then subjected to pulmonary challenge with 5 × 10^4^ CFUs wt *B. melitensis* 16M. Circulating lymphocytes were identified by *in vivo* IV labeling with an anti-CD45 mAb as per **Figure 3**. Flow cytometric analysis of isolated lung lymphocytes shows the circulating lymphocyte population identified as IV CD45^+^ cells on **(A)** day 56 and **(B)** day 84, gated as TCRβ^+^ cells, and then gated as CD4^+^ and CD8^+^ T-cell subsets. The total numbers of circulating lung **(C)** CD4^+^ and **(D)** CD8^+^ T cells on days 56 and 84 are depicted. Data are representative of three independent experiments with n ≥5 mice/group. Differences within T-cell subsets compared to PBS-dosed mice of that subset are shown: *p <0.05; **p <0.005, ***p <0.0005, and ****p <0.0001 using two-way analysis of variance performed followed by Tukey’s multiple comparisons test.

To characterize the types of immune T-cell responses responsible for vaccine-induced protection, the total T-cell numbers in the lungs and spleens pre- (day 56) and post-challenge (day 84) were examined using flow cytometry. Intravascular staining with fluorescent labeled anti-CD45 mAb permitted distinguishing between circulating (vascular) and noncirculating cell subsets. Within the noncirculating cells, lung CD4^+^ T cells induced by znBM-mC vaccination showed a >6-fold increase (p <0.0001) in total numbers compared to unvaccinated controls on day 56 (**Figures 3A, C**). Noncirculating lung CD8^+^ T cells showed a >8-fold increase (p <0.0001) in total number when compared to unvaccinated controls on day 56 (**Figures 3A, D**). Both noncirculating CD4^+^ and CD8^+^ T cells were nearly depleted after challenge, with total numbers approximating levels achieved in unvaccinated, challenged controls on day 84. While Rev 1-vaccinated mice showed increases in percentages for both CD4^+^ and CD8^+^ T cells on day 56 (**Figures 3A, B**), the increases in total numbers were not statistically significant **(** **Figures 3C, D**). For vascular T cells in the lungs, a significant increase in total numbers of CD4^+^ T cells was observed on day 56 by over 3-fold in Rev 1-vaccinated mice (p <0.05), and these were maintained post-challenge (**Figures 4A–C**). For znBM-mC-vaccinated mice, an increase in vascular CD4^+^ T cells was not statistically significant post-vaccination (**Figures 4A–C** **)**. A similar pattern was observed in the vascular CD8^+^ T cell subsets. Rev 1-vaccinated mice showed greater than a two-fold increase on day 56, and total numbers were maintained post-challenge. For znBM-mC-vaccinated mice, increases in the vascular CD8^+^ T cells in the lungs were not statistically significant **(** **Figures 4A, B, D**).

### Mucosal vaccination with znBM-mC induces increases in systemic CD4^+^ and CD8^+^ T-cell subsets

**Figure 5:**
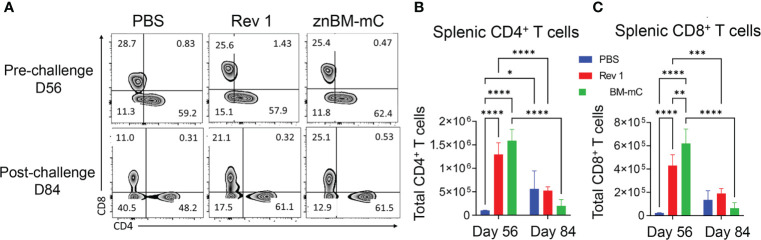
Mucosal vaccination with znBM-mC induces T-cell expansion in the spleens. Groups of mice were vaccinated with sPBS, Rev 1, or znBM-mC, and then subjected to pulmonary challenge with 5 × 10^4^ CFUs wt *B. melitensis* 16M as described in **Figure 2**. **(A)** Flow cytometric analysis of isolated splenic lymphocytes previously gated on singlets, CD45^+^, TCRβ^+^ cells, and then gated as CD4^+^ and CD8^+^ T-cell subsets. Total numbers of splenic **(B)** CD4^+^ T cells and **(C)** CD8^+^ T cells on days 56 (pre-challenge) and 84 (post-challenge) are shown. Data are representative of two independent experiments with n = 5–7 mice/group. Differences within T-cell subsets compared to PBS-dosed mice of that subset as well as differences between treatment groups at both timepoints analyzed are shown: *p <0.05; **p <0.005, ***p <0.0005, and ****p <0.0001. Two-way analysis of variance (ANOVA) followed by Tukey’s multiple comparisons test was used for statistical analysis.

In the spleens of znBM-mC-vaccinated mice (**Figure 5**), CD4^+^ and CD8^+^ T cell total numbers increased >10-fold for CD4^+^ and 20-fold for CD8^+^ T cells, respectively, when compared with PBS-dosed controls on day 56 (**Figures 5B, C** **)**. After the challenge on day 84, both T-cell subsets were significantly (p <0.005) reduced compared to pre-challenge levels and approximated T cells in the unvaccinated controls. Mucosal vaccination with the Rev 1 strain also induced significant T-cell expansion. CD4^+^ T cells increased more than 10-fold (p <0.0001) on day 56 and significantly decreased post-challenge. Similarly, splenic CD8^+^ T cells in Rev 1-vaccinated mice increased more than 7-fold post-vaccination (p <0.0001) compared to naive controls. This population also significantly decreased post-challenge as the total number was depleted.

### IFN-γ is required for protection in znBM-mC-vaccinated mice

**Figure 6:**
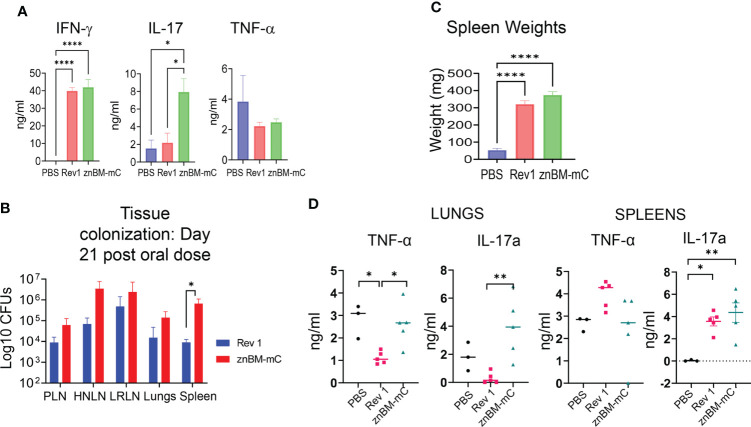
IFN-γ is required for vaccine clearance and predominates the proinflammatory cytokine response in the lungs post-vaccination. **(A)** Groups of BALB/c mice (5–7 mice/group) vaccinated with Rev 1 or znBM-mC or naïve controls using the previously described protocol, and evaluated for cytokine responses on day 56. Total lung mononuclear cells were restimulated *in vitro* with *Brucella* Ag for 72 h and cytokine ELISAs for IFN-γ, IL-17a, and TNF-α were performed on collected supernatants; ****p <0.0001, *p <0.05. **(B)** Tissue colonization of peripheral lymph nodes (PLNs), head and neck LNs (HNLNs), lower respiratory LNs (LRLNs), lungs, and spleens were evaluated 21 days after oral immunization of IFN-γ^−/−^ mice with Rev 1 or znBM-mC; *p <0.05. **(C)** The extent of splenic inflammation in IFN-γ^−/−^ mice was measured 21 days after oral vaccination with sPBS, Rev 1, or znBM-mC; ****p <0.0001 vs. PBS-dosed mice. **(D)** Cytokine levels in splenic and lung lymphocyte culture supernatants. Groups of IFN-γ^−/−^ mice (five mice/group) vaccinated with a single oral dose or sPBS, Rev 1, and znBM-mC as previously described protocol were euthanized on day 21. Splenic and lung lymphocytes were restimulated *in vitro* with *Brucella* Ag for 72 h and cytokine-specific ELISAs for IFN-γ, IL-17a and TNF-α were performed on collected supernatants. One-way ANOVA was used to compare group means. *p <0.05; **p <0.005. Experiment in **(A)** was repeated twice, and experiments in **(B–D)** were performed once.

Expression of specific effector functions such as proinflammatory cytokines is a measure of the quality of the vaccine-induced immune response and an important step in the characterization of immunogenicity (37, 46). After mucosal vaccination with Rev 1 or znBM-mC (oral prime-IN boost), isolated lung lymphocytes from these mice and naïve mice were restimulated *in vitro* with *Brucella* antigen for 72 h, and cytokine ELISAs were performed on collected supernatants to measure induced IFN-γ, IL-17a, and TNF-α. Although IFN-γ and TNF-α concentrations were elevated for both znBM-mC- and Rev 1-vaccinated mice, they did not differ from each other (**Figure 6A**). However, IL-17a was significantly greater by three-fold for the znBM-mC group than in Rev 1-vaccinated mice (**Figure 6A**).

To better understand the inflammatory response elicited by znBM-mC, studies were undertaken in IFN-γ^−/−^ mice. Groups of IFN-γ^−/−^ mice were orally dosed with the prescribed doses of znBM-mC, Rev 1, or sPBS on day 0. However, it was observed that the vaccinated mice died or reached the criteria for euthanasia before the experiment could be completed. Rev 1-vaccinated mice had a median survival time of 21 days post-dosing, whereas znBM-mC mice had a median survival time of 38 days. Simple survival analysis (Kaplan–Meier) identified significant differences between survival curves. (not shown). Since no adverse reactivity to the vaccines was observed by day 21 post-vaccination, a second group of mice were orally dosed with znBM-mC, Rev 1, or sPBS. On day 21 post-vaccination (before any significant illness was observed), peripheral lymph nodes (PLNs), head and neck LNs (HNLNs), lower respiratory LNs (LRLNs), lungs, and spleens were measured for the extent of brucellae colonization (**Figure 6B**). Quantification of tissue bacterial load revealed that both znBM-mC and Rev 1 disseminated throughout IFN-γ^−/−^ mice, resulting in vaccine colonization in various LNs, including PLNs, HNLNs, and LRLNs, as well as in the lungs and spleen. Splenic inflammation was noted with a more than 5-fold increase in splenic weights in both Rev 1- and znBM-mC vaccinated IFN-γ^−/−^ mice (**Figure 6C**). To measure the inflammatory cytokines in IFN-γ^−/−^ mice, TNF-α and IL-17a, cytokine-specific ELISAs were performed on supernatants collected from Ag-restimulated lung and splenic mononuclear cells. Although TNF-α levels were reduced for lung lymphocytes in Rev 1-vaccinated mice, lung lymphocytes from znBM-mC-vaccinated mice showed significantly increased IL-17a compared with Rev 1-vaccinated mice (**Figure 6D**). IL-17a was also enhanced in splenic supernatants from both Rev 1- and znBM-mC-vaccinated groups compared with PBS-dosed controls; splenic TNF-α levels did not differ among any group (**Figure 6D**).

### Mucosal vaccination with znBM-mC induces a lung Th1 cell response

**Figure 7:**
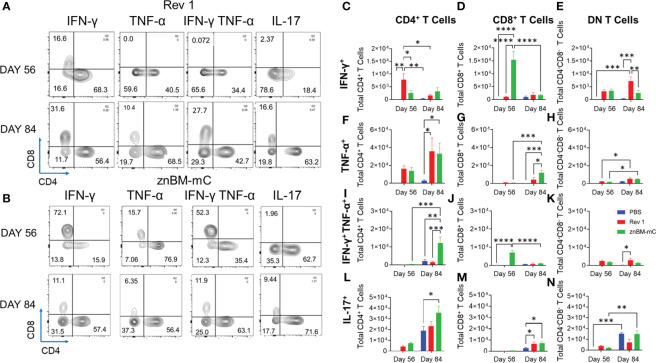
Mucosal vaccination with znBM-mC enhances cytokine production by lung CD4^+^ and CD8^+^ T cells. Groups of female BALB/c mice (10–14 mice/group) were vaccinated using the previously established protocol, and half the mice were subjected to pulmonary challenge with wt *B. melitensis* 16M as previously described. Pre- (day 56) and post-challenge (day 84) lung lymphocytes were isolated and restimulated overnight with the equivalent of 10^9^ CFUs/ml of *Brucella* Ag. Surface and intracellular staining were performed to measure the cytokine response. **(A)** Representative FACS plots show distribution of cytokines produced upon gating on cytokine^+^ TCRβ^+^ cells from Rev 1-vaccinated mice into CD4^+^ and CD8^+^ T cells on day 56 (top row) and day 84 (bottom row). **(B)** Representative FACS plots show distribution of cytokines produced upon gating on cytokine^+^ TCRβ^+^ cells from znBM-mC-vaccinated mice into CD4^+^ and CD8^+^ T cells on day 56 (top row) and day 84 (bottom row). Total numbers of cells producing IFN-γ for **(C)** CD4^+^ **(D)** CD8 ^+^ and **(E)** CD4^−^ CD8^−^ (double-negative; DN) T cells are depicted. Total numbers of TNF-α−producing **(F)** CD4^+^, **(G)** CD8^+^, and **(H)** DN T cells are shown. Total numbers of IFN-γ^+^ TNF-α^+^ **(I)** CD4^+^, **(J)** CD8^+^, and **(K)** DN T cells are depicted. Total numbers of IL-17-producing **(L)** CD4^+^, **(M)** CD8^+^, and **(N)** DN T cells are shown. The data depict the sum of two experiments, and two-way analysis of variance followed by Tukey’s multiple comparisons test was performed; *p <0.05, **p <0.005, ***p <0.0005, ****p <0.0001 vs. PBS-dosed mice.

Flow cytometry analysis was performed to measure the extent of the vaccine-induced Th1 cell response. Prior to termination, circulating lymphocytes were *in-vivo* labeled by a retro-orbital injection of anti-CD45 mAb. Isolated lung lymphocytes were restimulated overnight with *Brucella* Ag to measure activation status and cytokine production. Prior to challenge, both Rev 1- and znBM-mC-vaccinated mice showed similar distributions of lung vascular and noncirculating IFN-γ- and IL-17-producing T cells with slightly less lung vascular TNF-α-producing T cells by znBM-mC-vaccinated mice (**Supplementary Figure 1A**). Interestingly, about one-third of the IL-17^+^ noncirculating lung cells were not T cells. Four weeks after challenge (day 84), one striking difference found between Rev 1- and znBM-mC-vaccinated mice was noted. The majority of the lung vascular IFN-γ-producing cells was derived from T cells in znBM-mC-vaccinated as opposed to only about two-thirds from T cells and one-third from non-T cells in Rev 1-vaccinated mice (**Supplementary Figure 1B**). The majority of the lung vascular IL-17-producing cells were T cells in znBM-mC-vaccinated mice post-challenge. For noncirculating cells, the relative percentages of IFN-γ-, TNF-α-, and IL-17-producing cells post-challenge were similarly distributed between T cell and non-T cell subsets for both Rev 1- and znBM-mC-vaccinated mice (**Supplementary Figure 1B**). On day 56, no cytokine production was detected in the lungs of PBS-dosed mice prior to challenge. On day 84, PBS-dosed mice showed cytokine production with vascular IFN-γ being produced by mostly T cells and, and over three-quarters vascular TNF-α and IL-17 produced by T cells. IFN-γ, TNF-α, and IL17 are mostly derived from noncirculating T cells (**Supplementary Figure 1B**). Further analysis of total lung T cells at pre- (day 56) and post-challenge (day 84) revealed that the noncirculating T cells for both Rev 1- and znBM-mC-vaccinated mice contained the majority of cytokine-producing T cells (**Supplementary Figure 2**). Hence, the noncirculating T cells were further analyzed to assess the source of these cytokine-secreting cells **(** **Figure 7** **)**. Gating on TCRβ^+^ cells, the ratio of CD4^+^:CD8^+^ T cells was measured as well as their polyfunctional capacity **(** **Supplementary Figures 3A–F** **).** Upon examination of resident (IV CD45^-^) lung T cells from Rev 1-vaccinated mice, the majority of IFN-γ, both pre- (day 56) and post-challenge (day 84), were contained within CD4^+^ T cells (**Figures 7A, C–E**). In this group, IFN-γ-producing CD4^+^ T cells were significantly greater than those in PBS-dosed mice on day 56 (p <0.005) (**Figures 7A, C**). In contrast, CD8^+^ T cells made up the majority of IFN-γ producing cells in znBM-mC-vaccinated mice on day 56 increasing over 12-fold compared to PBS-dosed mice (p <0.0001) (**Figures 7B, E**). However, by day 84 (when total T-cell populations declined), the majority of IFN-γ producing CD4^+^ and CD8^+^ T cells were diminished for both Rev 1- and znBM-mC-vaccinated groups. At this timepoint, a significantly higher number of double negative (DN)—CD4^−^ CD8^−^ T cells expressing IFN-γ was detected in Rev 1-vaccinated mice compared to both PBS-dosed and znBM-mC-vaccinated mice (**Figures 7A, B, E**). Differences in TNF-α production by the T-cell subsets were also evident. While there was no significant difference in total number of any TNF-α producing T-cell subset and PBS-dosed controls on day 56, total numbers increased significantly for both Rev 1- and znBM-mC-vaccinated mice relative to PBS-dosed controls on day 84 (**Figures 7A, B, F–H**). Similar numbers of CD4^+^ and DN T cells produced TNF-α in both Rev 1 and znBM-mC groups, while significantly more (2-fold) CD8^+^ T cells produced TNF-α in znBM-mC vaccinated mice compared to Rev 1-vaccinated mice (p <0.05) (**Figures 7A, B, G**). Polyfunctional T cells producing both IFN-γ and TNF-α yielded interesting results. On day 56 in znBM-mC-vaccinated mice, CD8^+^ T cells dominated, while Rev 1-vaccinated mice had no polyfunctional CD8^+^ T cells (p <0.0001) (**Figures 7A, B, I–K**). Instead, Rev 1 polyfunctional T cells were limited to CD4^+^ and DN T cell subsets on day 56 (**Figures 7A, B, I–K**). On day 84, Rev 1-vaccinated mice showed a significant increase in total numbers of polyfunctional DN T cells (p <0.05), and an over 4-fold increase in CD4^+^ polyfunctional T cells (p <0.005) (**Figures 7A, B, I–K**). On day 56, IL-17 production by lung resident T cells was derived from CD4^+^ T cells and a DN T cell population in both Rev 1- and znBM-mC-vaccinated mice. No significant difference in total cell numbers was detected for either T-cell subset (**Figures 7A, B, L–N**). On day 84, total numbers of IL-17-expressing CD4^+^ T cells were significantly greater for znBM-mC-vaccinated mice than for PBS-dosed controls (p <0.05). Both Rev 1- and znBM-mC-vaccinated mice showed significant increase in total numbers of IL-17-expressing CD8^+^ T cells compared with PBS-dosed controls, and no difference in total numbers of IL-17-producing DN T-cell subsets (**Figures 7A, B, L–N**).

### Mucosal vaccination with znBM-mC induces both non-circulating memory CD4^+^ and CD8^+^ T cells exhibiting either effector or resident memory phenotype

**Figure 8:**
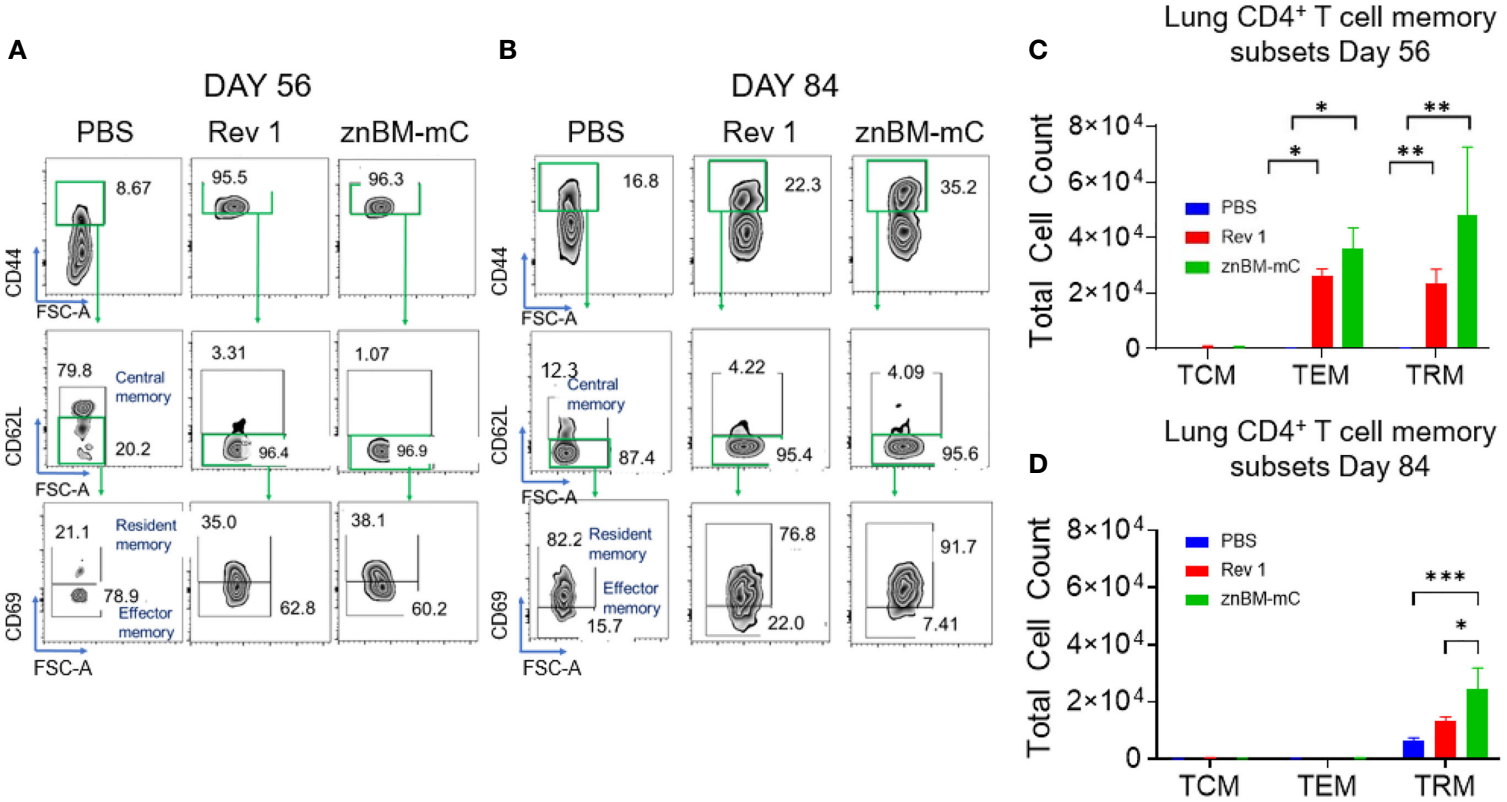
Mucosal vaccination with znBM-mC induces lung memory CD4^+^ T cells comprised of both TEMs and TRMs. Groups of BALB/c mice (10-14/group) vaccinated with PBS, Rev 1, or znBM-mC, and then challenged as previously described. **(A)** Pre- (day 56) and **(B)** post-challenge (day 84) lung lymphocytes were isolated and restimulated with Ag overnight as described in **Figure 7**. FACS plots depict (A) day 56 and (B) day 84 noncirculating memory lung CD4^+^ T cells from mice vaccinated with PBS, Rev 1, or znBM-mC. **(C)** Total numbers of pre-challenge and **(D)** post-challenge lung CD4^+^ TCM, TEM, and TRM subsets are depicted. Data are representative of two experiments. Two-way analysis of variance followed by Turkey’s multiple comparisons test was performed; *p < 0.05, **p < 0.005, and ***p < 0.0005 vs. PBS-dose mice.

Memory T cells are distinguished by their location: central memory T cells (TCMs) located in the peripheral lymph nodes; effector memory T cells (TEMs) and resident memory T cells (TRMs) located in peripheral tissues such as the lungs (47–49). The noncirculating (IV CD45^−^) memory CD4^+^ T cells in both pre- and post-challenged lungs contained TEMs evident by their CD44^hi^, CD62L^lo^, CD69^-^ expression, and TRMs by their CD44^hi^, CD62L^lo^, CD69^+^ expression (**Figures 8A–D**). TCMs, recognized as CD44^hi^ and CD62L^+^, were negligibly induced in the lungs by Rev 1 or znBM-mC either on day 56 or 84. Both CD4^+^ TEMs (p <0.05) and CD4^+^ TRMs (p <0.005) for Rev 1- and znBM-mC-vaccinated groups were significantly increased on day 56 compared with PBS-dosed controls (**Figure 8C**). PBS-dosed mice showed minimal memory of CD4^+^ T-cell subsets. After the challenge on day 84, the CD4^+^ TEMs were mostly depleted for both Rev 1- and znBM-mC-vaccinated mice, and the remaining CD4^+^ T cells were mostly TRMs (**Figures 8B, D**). CD4^+^ TRMs were slightly induced in PBS-dosed, *B. melitensis* 16M-challenged mice, and znBM-mC-vaccinated mice showed more than three-fold more CD4^+^ TRMs in comparison, and they were also significantly greater than those in Rev 1-vaccinated mice (p <0.05) (**Figure 8D**). This evidence demonstrates the longevity of the TRMs within the lungs.

**Figure 9:**
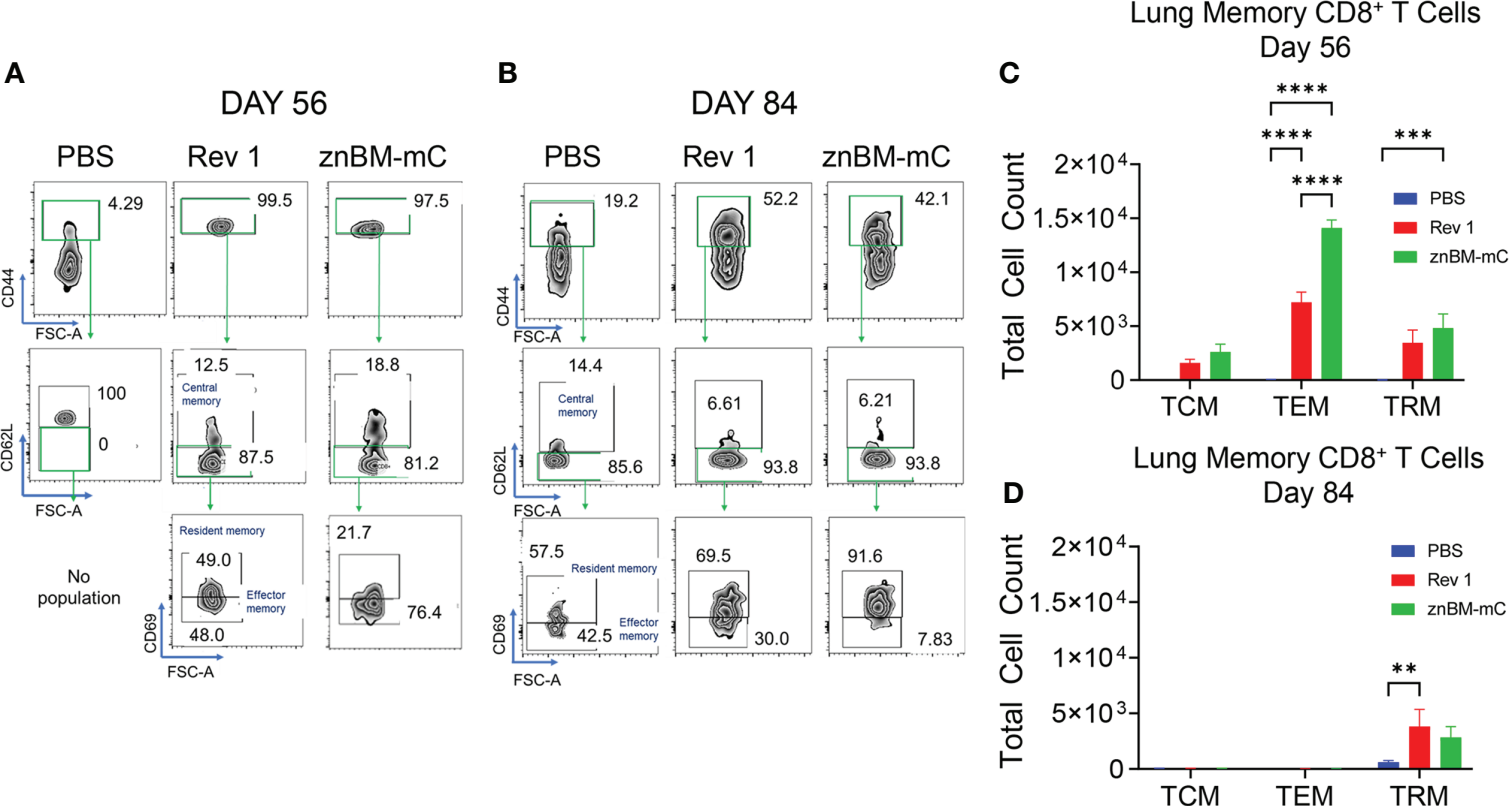
Mucosal vaccination with znBM-mC induces nonrecirculating lung memory CD8^+^ T cells comprised of TCMs, TEMs, and TRMs. Groups of BALB/c mice (10–14/group) were vaccinated and challenged as described in **Figure 8**. **(A)** Pre- (day 56) and **(B)** post-challenge (day 84) lung lymphocytes were isolated and restimulated with Ag overnight as described in **Figure 7**. FACS plots depict **(A)** day 56 and **(B)** day 84 noncirculating memory lung CD8^+^ T cells from mice vaccinated with sPBS, Rev 1, or znBM-mC. **(C)** Total numbers of pre-challenge and **(D)** post-challenge non-circulating lung CD8^+^ memory T-cell subsets are shown. Data are representative of two experiments: **p <0.005, ***p <0.0005, and ****p <0.0001 vs. PBS-dosed mice.

CD8^+^ T-cell subsets are also similarly classified as TCMs, TEMs, and TRMs as described for CD4^+^ T cells. Gating on the in IV CD45^-^ TCRβ^+^ cells, CD8^+^ TCMs, TEMs, and TRMs were identified (**Figure 9**). On day 56, these memory T-cell subsets were all present in the lungs of Rev 1- and znBM-mC-vaccinated mice with CD8^+^ TEMs predominating (**Figures 9A, C**). No memory CD8^+^ T cells were detected in PBS-dosed mice prior to challenge (day 56). The total numbers of CD8^+^ TEMs were twice greater in znBM-mC-vaccinated mice than in Rev 1-vaccinated mice (p <0.0001). At this timepoint, CD8^+^ TRMs from znBM-mC-vaccinated mice were significantly greater than those in PBS-dosed and Rev 1-vaccinated mice (p <0.005). As observed for CD4^+^ TEMs, CD8^+^ TEMs, and TCMs were mostly depleted by day 84, and the remaining CD8^+^ T cells were nearly all TRMs (**Figures 9B, D**). Both the Rev 1- and znBM-mC-vaccinated groups showed more than three-fold greater numbers than the PBS-dosed controls.

### Lung protection induced by mucosal vaccination with znBM-mC is maintained in the absence of CD4^+^ or CD8^+^ T cells or IL-17

**Figure 10:**
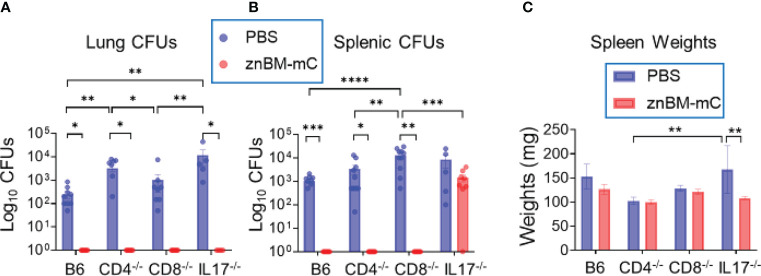
Mucosal vaccination with znBM-mC protects CD8^−/−^, CD4^−/−^, and IL17^−/−^ mice from pulmonary challenge with wt *B. melitensis* 16M. Groups of C57BL/6 (B6), CD4^−/−^, CD8^−/−^, and IL-17^−/−^ mice were either dosed with sPBS or vaccinated with znBM-mC as previously described. On day 56, all mice were subjected to a pulmonary challenge with wt *B. melitensis* 16M as previously described. Two weeks after challenge, lungs and spleens were harvested to measure the extent of brucellae colonization. **(A)** Lung colonization in all mouse strains post-challenge with wt *B. melitensis* 16M of all four mouse strains vaccinated with sPBS or znBM-mC. **(B)** Splenic colonization in all mouse strains post-challenge with wt *B. melitensis* 16M. **(C)** Splenic weights of all sPBS-dosed or znBM-mC-vaccinated mice for the different mouse strains are depicted. Two-way analysis of variance and unpaired t-tests were used for statistical analysis: *p <0.05, **p <0.005, ***p <0.0005, and ****p <0.0001 versus the indicated group. This experiment was performed once with 10 mice/group.

Past studies have identified either CD4^+^ or CD8 T^+^ cells as being responsible for protection against *Brucella* infections (25, 35, 37, 46, 50). Although IFN-γ was produced by CD4^+^, CD8^+^, and DN T cells, some differences were observed among the IFN-γ-producing T cells for Rev 1- and znBM-mC-vaccinated mice. Additionally, IL-17 was mostly derived from lung CD4^+^ T cells. To determine if protection was dependent upon CD4^+^, CD8^+^, or IL-17^+^ T cells, groups of CD8^−/−^, CD4^−/−^, and IL-17^−/−^ mice on a B6 background were vaccinated with znBM-mC as previously described. B6 (wt) mice were similarly vaccinated as a control, and PBS-dosed controls for each mouse strain were included. All mice were subjected to pulmonary challenge on day 56 post-primary vaccination, and two weeks post-challenge, the study was terminated, and harvested spleens and lungs were examined for the extent of brucellae colonization. All CD4^−/−^, CD8^−/−^, and IL-17^−/−^ mice showed equivalent protection in the lungs as wt B6 mice (**Figure 10A**). Likewise, CD4^−/−^ and CD8^−/−^ spleens from mice vaccinated with znBM-mC showed sterile protection from virulent *B. melitensis* 16M challenge as did vaccinated B6 mice. However, znBM-mC-vaccinated IL-17^−/−^ mice showed no significant difference in splenic colonization compared to PBS-dosed IL-17^−/−^ controls against the wt *B. melitensis* 16M challenge (**Figure 10B**). Some differences in wt *B. melitensis* 16M colonization were observed in different mouse strains of PBS-dosed mice. In the lungs of PBS-dosed mice, the CD4^−/−^ and IL-17^−/−^ groups showed significantly higher bacterial colonization than B6 and CD8^−/−^ mice by more than one-log increase in brucellae load; no significant difference between B6 and CD8^−/−^ mice was observed (**Figure 10A**). Conversely, in the spleens, while B6, CD4^−/−^ and IL-17^−/−^ mice showed similar levels of splenic brucellae colonization, PBS-dosed CD8^−/−^ mice showed a significant increase in brucellae colonization by one-log (**Figure 10B**). Splenic weights for PBS-treated or vaccinated CD4^−/−^ and CD8^−/−^ mice did not significantly differ nor from PBS-treated B6 mice (**Figure 10C**). However, znBM-mC-treated IL-17^−/−^ mice did show a significant decrease in splenic weight compared to PBS-dosed IL-17^−/−^ mice (**Figure 10C**).

## Discussion

Most infectious diseases result after crossing a mucosal barrier. Although brucellosis is frequently described as a disease of the reproductive tract in livestock (1, 2, 18, 51), transmission usually occurs following oral or aerosol exposure in humans (12, 33, 52). *Brucella* is an intracellular pathogen and, regardless of its route of exposure, *Brucella* will always disseminate systemically (53, 54). Thus, cell-mediated immunity must mitigate the infection (11, 21, 35, 46, 50, 55). Given this latter attribute, current livestock vaccines are administered parenterally, and few studies have examined the potential of mucosal vaccination to establish protective immunity for both mucosal and systemic compartments (12, 37). As described here, an optimized mucosal vaccination strategy of oral prime and nasal boost with the double mutant, znBM-mC, proved best to confer protective immunity against a virulent pulmonary *B. melitensis* challenge. While nasal priming, nasal boost, or a single nasal dose also proved highly useful, systemic protection was not as effective. Establishing cell-mediated immunity at potential sites of exposure can aid in allaying infection and subsequent brucellae dissemination (51).

The efficacy induced by znBM-mC vaccination was compared to the efficacy elicited by the three standard livestock vaccines. When mice were orally primed and nasally boosted, both the znBM-mC and *B. melitensis* Rev 1 vaccines proved equally effective after two doses. Oral prime, nasal boost of mice with *B. abortus* RB51 vaccine proved ineffective against pulmonary challenge with wt *B. melitensis* 16M, while mice vaccinated by the same routes with *B. abortus* S19 proved effective in the lungs but not in the spleen. The results with RB51 are consistent with the effectiveness against wt *B. melitensis* 16M challenges in previous studies (25, 29) and with wt *B. abortus* 2308 challenges (37, 40). The results for the S19-vaccinated mice are also similar to those obtained against the wt *B. abortus* 2308 challenge (39). Thus, znBM-mC elicits exquisite protection against a virulent *B. melitensis* 16M challenge with less splenic inflammation than Rev 1. While detailed safety studies were not performed, the lack of adverse clinical symptoms post-vaccination, the absence of vaccine persistence both locally and systemically, and the lack of splenomegaly indicate that mucosal vaccine administration is safe in this model.

In addition, Th1 cell immunity is essential for protection against *Brucella* infection, and in its absence, mice endure greater tissue colonization (29, 32, 34, 35). In this study, we noticed an interesting pattern: whereas the significant increase in lung T cells was more evident in the noncirculating population for znBM-mC vaccinated mice, the opposite was noticed for Rev-1-vaccinated mice, where the significant increase in total T-cell numbers was noted for the circulating population. Nevertheless, in both Rev 1- and znBM-mC-vaccinated mice, the majority of cytokine producing T cells were located in within noncirculating compartment. Additionally, within the circulating lung T cells, IFN-γ and TNF-α cytokine production was not exclusive to T cells in Rev 1-vaccinated mice post-challenge, whereas in znBM-mC-vaccinated mice, T cells produced the majority of proinflammatory cytokines at this timepoint. The significant increase in noncirculating T-cell populations in znBM-mC-vaccinated mice resulted in these mice showing significantly higher numbers of differentiated noncirculating memory T-cell populations prior to challenge. The differentiation of both CD4^+^ and CD8^+^ T cells into memory subsets showed a similar pattern, albeit different total numbers among both vaccinated groups. However, both maintained their Trm populations and showed similar protection after challenge. Evaluation of the cytokine profiles of the recruited CD4^+^ and CD8^+^ T cells revealed that at pre-challenge, the majority of IFN-γ was derived from CD8^+^ T cells and much less by CD4^+^ T cells in the lungs of znBM-mC-vaccinated mice. Additionally, the polyfunctional CD8^+^ T cells were also increased in znBM-mC-vaccinated mice. In contrast, lung IFN-γ-producing cells in Rev 1-vaccinated mice mostly associated with CD4^+^ T cells. Examination of cytokine-producing T cells post-challenge revealed that both TNF-α^+^ and polyfunctional CD4^+^ T cells were increased 4 weeks post-challenge but not for IFN-γ^+^, TNF-α^+^, or polyfunctional CD8^+^ T cells. At this same time point, IFN-γ^+^, TNF-α^+^, or polyfunctional CD4^+^ and CD8^+^ T cells remained reduced in Rev 1-vaccinated mice. Moreover, some have suggested that timing of IFN-γ induction is more relevant for later response in infection and indispensable for earlier time points (56, 57). In this study, IFN-γ was deemed essential early in the response since IFN-γ^−/−^ mice were unable to clear znBM-mC nor Rev 1 vaccine, which is consistent with what others have shown, *Brucella* being lethal in IFN-γ^−/−^ mice (32, 34).

Though less characterized, Th17 cell responses have been implicated in immunity to brucellosis. Studies using the murine model of infection suggest that while Th17 cell responses are induced during wt *Brucella* infection, they do not have a role in the clearance of wt brucellae (27). Interestingly, in our study, IL-17^−/−^ mice dosed with PBS and challenged with wt *B. melitensis* did not show significant differences in splenic colonization. However, lung colonization was significantly increased during *B. melitensis* infection compared with B6 mice. Past studies examining the role of IL-17 in the vaccine-induced protective response suggest that Th17 cells can contribute. Oral immunization with unlipidated *Brucella* Omp19 stimulated IL-17, and *in vivo* neutralization of IL-17 resulted in reduced efficacy (27). In a similar vein, oral or nasal vaccination with Δ*znuA B. melitensis* mutant stimulated Th17 cell responses, and upon its *in vivo* neutralization, reduced efficacy was observed (25, 29). In the current study, znBM-mC did induce Th17 cells in the lungs of vaccinated mice at a level significantly greater than either PBS-dosed wt mice or Rev 1-vaccinated controls in IFN-γ^−/−^ mice. In a previous study in our lab, increased IL-17 production was also elevated when using znBAZ, but IL-17 neutralization did not have any significant impact on tissue colonization nor vaccine efficacy (37). Given the level of IL-17 induction by znBM-mC, additional studies were pursued using IL-17^−/−^ mice. Although znBM-mC-vaccinated IL-17^−/−^ mice completely cleared the wt challenge strain of brucellae from the lungs, systemic abatement of the brucellae failed, as evidenced by the extent of colonization of the spleens. Hence, IL-17 is essential for systemic protection. Such a finding suggests there may be subtle differences in protective correlates between *B. abortus* and *B. melitensis* infections.

Protection against brucellosis is long believed to be CD4^+^ T cell-dependent and less on CD8^+^ T cells (35, 46, 58). Yet, brucellosis patients have been shown to exhibit increased levels of IFN-γ-producing CD8^+^ T cells (59, 60). The predilection for CD8^+^ T cell dependency may be linked to mucosal vaccinations since prior work with Δ*znuA B. melitensis* and znBAZ mutants has shown similar reliance (25, 37). Alternatively, vaccine formulation may influence the expansion and differentiation of T-cell subpopulations (40, 58). The relative contribution of whether resident or vascular T-cell subsets induced for protection against secondary *Brucella* infection have a role has yet to be extensively studied outside of our group. The results showed that the total numbers of T cells (both CD4^+^ and CD8^+^ subsets) and total numbers of cytokine-producing T cells are greater for the noncirculating lung T cells compared to circulating T cells. Previous investigation into the role of TRMs in the lungs revealed strikingly elevated CD8^+^ TRMs in znBAZ-vaccinated mice (37). The results from the current study show that mucosal znBM-mC vaccination induced significant expansion of both CD4^+^ and CD8^+^ memory T-cell subsets in the lungs of both effector and long-lived resident phenotypes. Additionally, in the spleens of the znBM-mC-vaccinated mice, both CD4^+^ and CD8^+^ T cells significantly increased. When further evaluated as to which memory T cells bore the source of pre- and post-challenge proinflammatory cytokines, the noncirculating IFN-γ^+^ T cells exceeded vascular IFN-γ^+^ T cells for both Rev 1- and znBM-mC-vaccinated mice. Likewise, noncirculating TNF-α^+^ and IL-17^+^ T cells were augmented relative to those present in the vascular compartment. Thus, successful protection against a virulent *B. melitensis* challenge may be linked to the capacity to induce proinflammatory TRMs.

Cytokines induced by znBM-mC versus Rev 1 vaccination showed a preferential bias for IFN-γ-producing CD8^+^ T cells and IFN-γ-producing CD4^+^ T cells, respectively. Interestingly, though, such bias was not predictive of which T-cell subset was essential for protection. Previous work with the *B. abortus* mutant, znBAZ, did show an IFN-γ-producing CD8^+^ T-cell bias, and immune protection was abated in CD8-deficient mice. In contrast, although znBM-mC vaccination also showed an IFN-γ-producing CD8^+^ T-cell bias, this response did not translate into similar protective immunity loss in CD8-deficient mice. In fact, immune protection was retained in either CD4^−/−^ or CD8^−/−^ mice.

In summary, mucosal vaccination with the live, attenuated *B. melitensis* mutant, znBM-mC, conferred protection in mice against pulmonary challenge with wt *B. melitensis* 16M. As with Rev 1, immune protection was T-cell-dependent, though differences in cytokine-producing T-cell subsets and patterns of T-cell expansion and location were noted. znBM-mC vaccination induced a robust Th1-type immunity by distinct memory subsets, including long-lived noncirculating memory T-cell subsets in the lungs, and IL-17 was found to be important for controlling systemic infection.

## Data availability statement

The original contributions presented in the study are included in the article/**Supplementary Material**. Further inquiries can be directed to the corresponding author.

## Ethics statement

This study was reviewed and approved by the University of Florida Institutional Animal Care and Use Committee.

## Author contributions

Conceptualization: ZG, XY, and DWP. Formal analysis: ZG, CH, and DWP. Funding acquisition: DWP. Investigation: ZG, XY, CH, and DWP. Methodology: ZG, XY, CH, and DWP. Validation: ZG, XY, CH, and DWP. Visualization: ZG, XY, CH, and DWP. Writing: ZG, XY, CH, and DWP. All authors contributed to the article and approved the submitted version.

## Funding

This work was supported by the National Institute of Allergy and Infectious Diseases grants AI123244 and AI125516 (DP).

## Conflict of interest

The authors declare that the research was conducted in the absence of any commercial or financial relationships that could be construed as a potential conflict of interest.

## Publisher’s note

All claims expressed in this article are solely those of the authors and do not necessarily represent those of their affiliated organizations, or those of the publisher, the editors and the reviewers. Any product that may be evaluated in this article, or claim that may be made by its manufacturer, is not guaranteed or endorsed by the publisher.
