# Supplementary material for: Live mucosal vaccination stimulates potent protection via varied CD4+ and CD8+ T cell subsets against wild-type Brucella melitensis 16M challenge
Source: Front Immunol. 2022 Oct 3;13:995327. doi: 10.3389/fimmu.2022.995327 (PMC9574439; doi:10.3389/fimmu.2022.995327)
Supplement: Supplementary file 1 [file DataSheet_1.pdf]

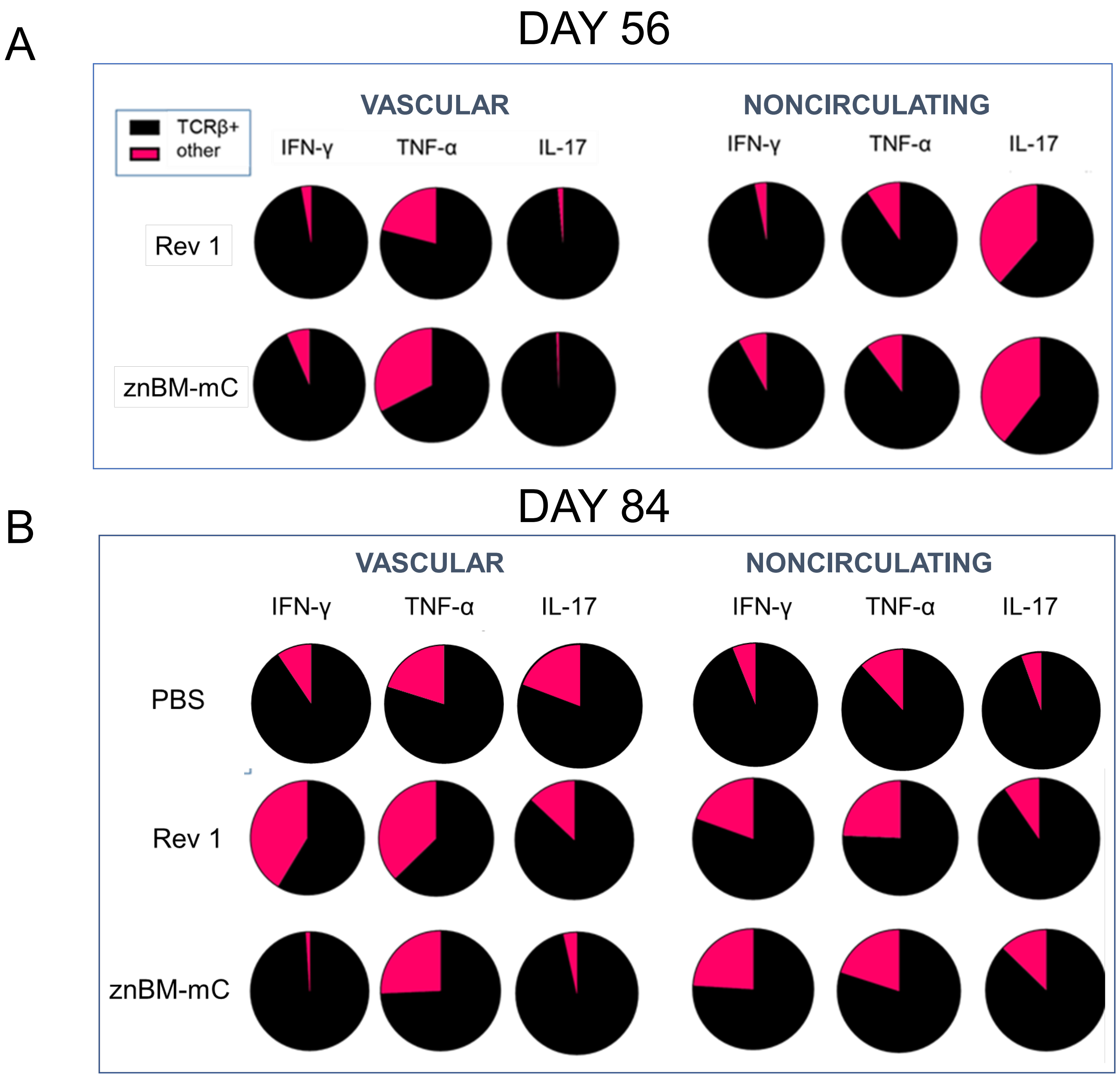

Supplemental Figure 1. After vaccination with Rev 1 and znBM-mC, lung T cells are the primary producers of proinflammatory cytokines in vascular and noncirculating compartments. Distribution of cytokine-producing lung lymphocytes pre-gated for lymphocytes and TCR-β^{+} and then gated as IV $CD45^{+}$ for vascular T cells or IV $CD45^{-}$ for noncirculating T cells and cytokine of interest. (A) Day 56 post-primary vaccination and (B) four weeks (Day 84) after vaccinated mice were challenged by the pulmonary route with wt *B. melitensis* 16M.

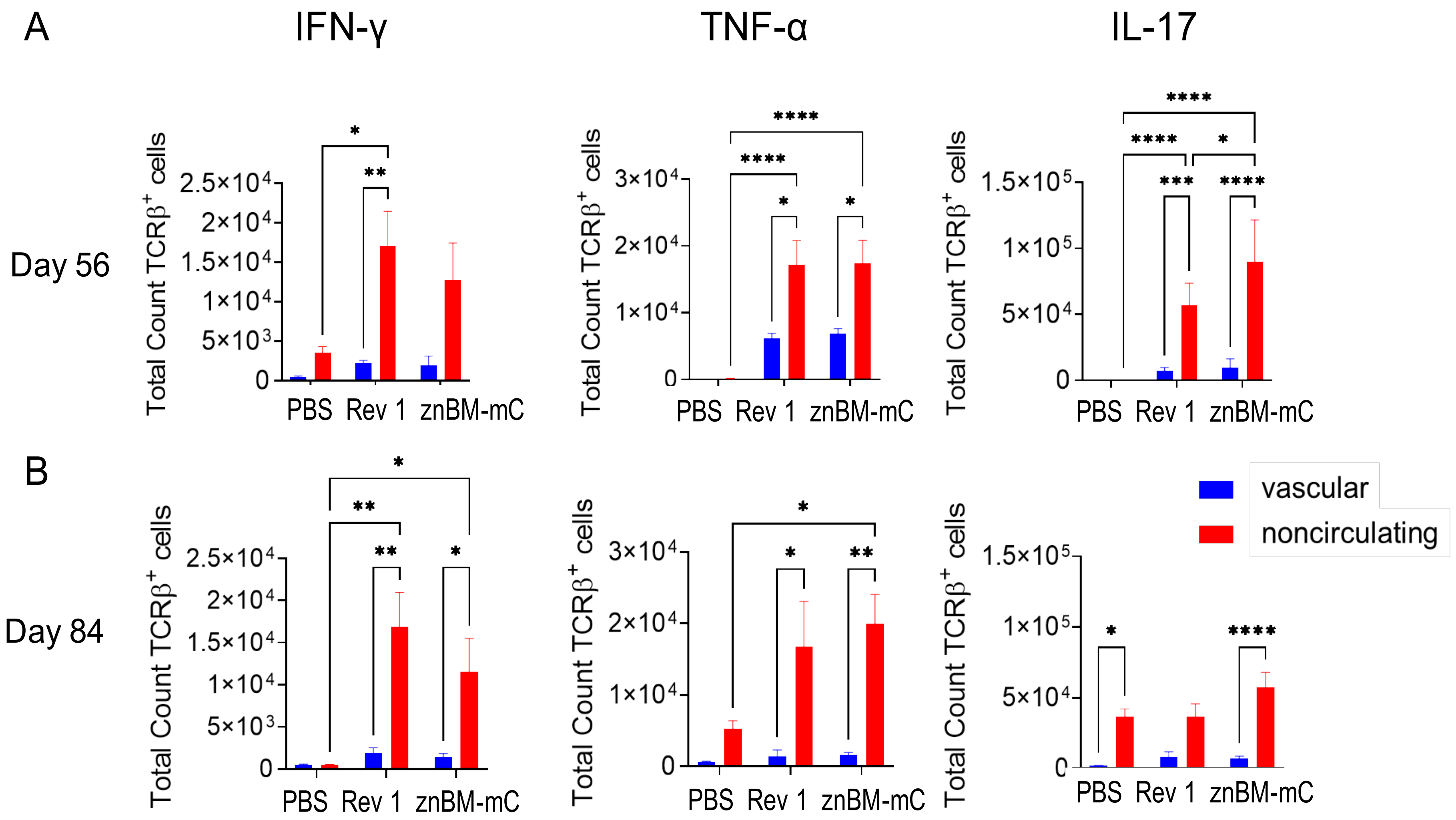

Supplemental Figure 2. Noncirculating lung T cells are the primary producers of lung proinflammatory cytokines in Rev 1- or znBM-mC-vaccinated mice. Total numbers of cytokine$^+$ TCRβ$^+$ cells in the lungs were identified by prior gating on lymphocytes, singlets and circulating/noncirculating CD45 population. Within each compartment, cytokine positive populations were gated to identify TCRβ$^+$ cytokine producing cells. (A). Total numbers of vascular/circulating and noncirculating lung T cells producing proinflammatory IFN-γ, TNF-α, or IL-17 on day 56 post-primary vaccination are shown. (B) Total numbers of vascular and noncirculating lung T cells producing IFN-γ, TNF-α, and IL-17 on day 84 (post-challenge) are shown; two-way ANOVA was used to compare populations: *$p < 0.05$, **$p < 0.005$, ***$p < 0.0005$, ****$p < 0.0001$.

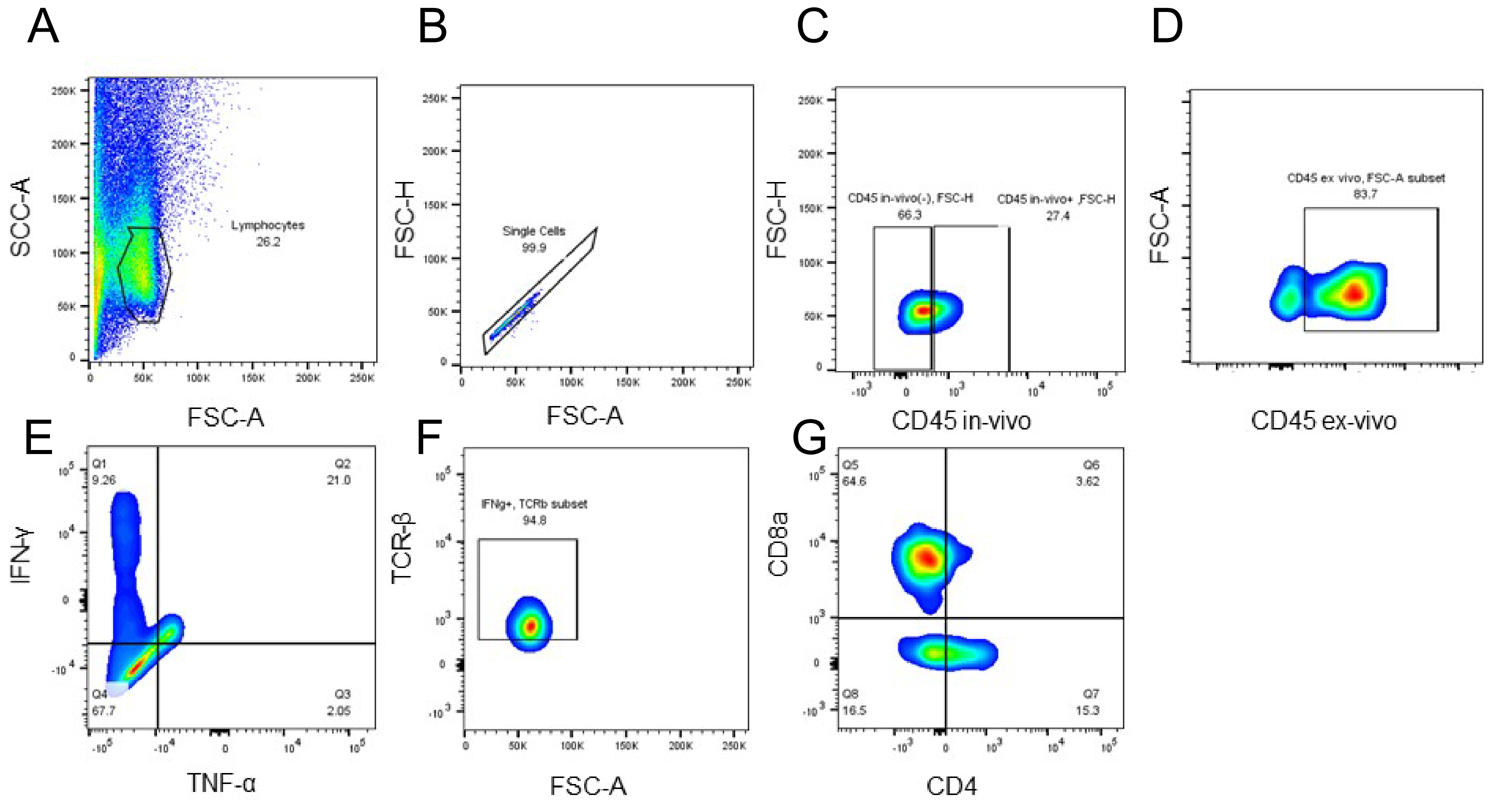

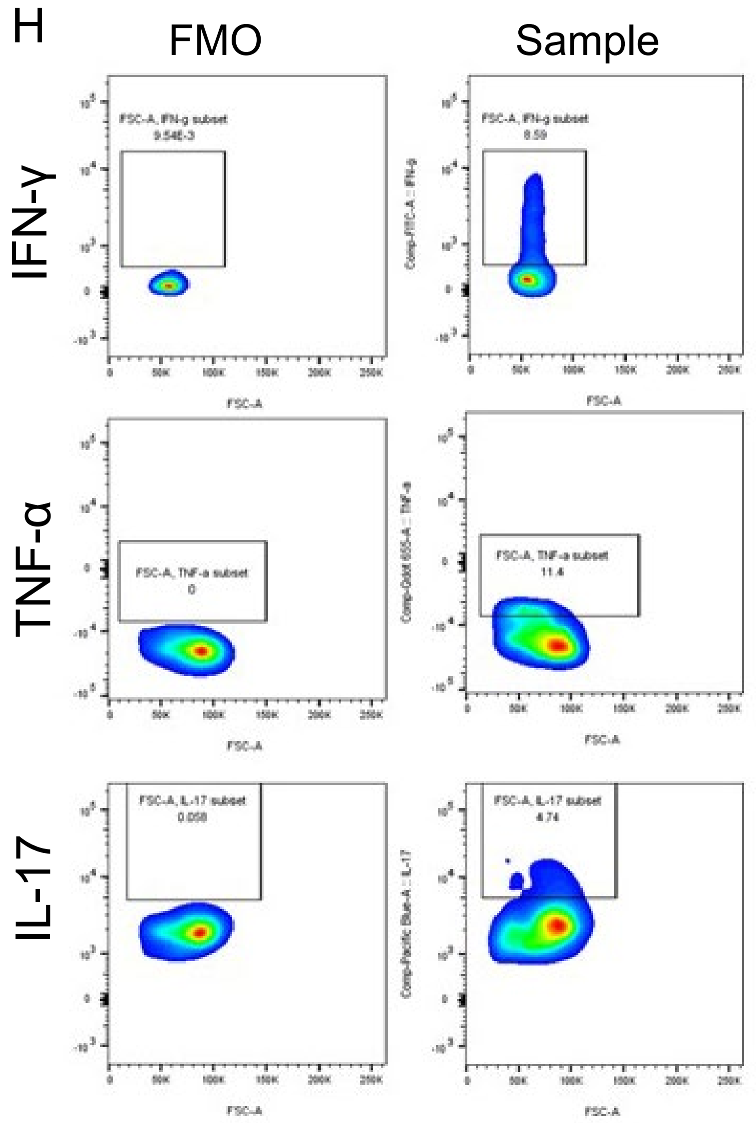

Supplemental Figure 3. Gating strategy used to identify T cell subset distribution for proinflammatory cytokine producing cells. Lymphocyte populations were identified and gated based of forward and side scatter (A). Doublets were excluded and single cell population was isolated based on height (FSC-H) and area (FSC-A) (B). Within the singlet population, circulating lymphocytes were separated based on the in vivo CD45 labeled cells (C). Noncirculating lymphocytes were further gated to separate the CD45 (ex-vivo labelled) compartment (D). Both circulating and noncirculating lymphocytes were gated on cytokine of interest. Polyfunctional T cells were identified by dual expression of IFN-γ and TNF-α (E). T cells within the cytokine positive populations were identified by TCR-β expression (F), and T cell subsets were defined by CD4 and CD8 expression (G). FMO controls were used to ensure proper gating of cytokine positive populations (H).
